# Retraction note: Moderate protection from vaccination against influenza A(H3N2) subclade K in Beijing, China, September to December 2025 (Euro Surveill. 2026;31(2))

**DOI:** 10.2807/1560-7917.ES.2026.31.7.260219re

**Published:** 2026-02-19

**Authors:** Eurosurveillance editorial team

**Affiliations:** 1 European Centre for Disease Prevention and Control (ECDC), Stockholm, Sweden

The *Eurosurveillance* editor-in-chief, in agreement with the authors, retracts the article "Moderate protection from vaccination against influenza A(H3N2) subclade K in Beijing, China, September to December 2025" by Shen et al, originally published on 15 January 2026 [1].

This retraction follows Committee on Publication Ethics (COPE) guidelines [2] and supersedes the expression of concern [3] that was published for this article on 22 January 2026 after the corresponding author had informed *Eurosurveillance* of a data handling error in the inclusion of influenza-like illness cases which may have led to biased influenza vaccine effectiveness estimates. 

An in-depth consultation with influenza experts on the *Eurosurveilllance* editorial board and guidance from two experts on publication ethics confirmed that the changes following recalculations have a major bearing on the interpretation of the findings, and a retraction of the article is necessary. We are grateful to the authors for flagging this honest error in a timely manner and for their smooth collaboration in resolving the issue.

Following these consultations, the editor-in-chief further agreed that the authors submit a new article with newer data and an analysis eliminating the error. That new manuscript has passed editorial evaluation and peer review and is published in Euro Surv. 2026;31(7) in the same issue as this Retraction note [4].
